# Chitosan Hydrogel as siRNA vector for prolonged gene silencing

**DOI:** 10.1186/1477-3155-12-23

**Published:** 2014-06-19

**Authors:** Zhiwei Ma, Chuanxu Yang, Wen Song, Qintao Wang, Jørgen Kjems, Shan Gao

**Affiliations:** 1 State Key Laboratory of Military Stomatology, Department of Periodontology and Oral Medicine, The School of Stomatology, Fourth Military Medical University, Xi-an, China; 2 Interdisciplinary Nanoscience Center (iNANO), Department of Molecular Biology and Genetics, Aarhus University, Gustav Wiedsvej 14, 8000 Aarhus C, Denmark; 3 State Key Laboratory of Military Stomatology, Department of Prothodontics, The School of Stomatology, Fourth Military Medical University, Xi-an, China

## Abstract

**Background:**

The periodontitis is one of the most prevalent diseases with alveolar resorption in adult people and is the main cause of the tooth loss. To investigate the possibility for protecting the loss of alveolar bone in periodontal diseases, a RNAi-based therapeutic strategy is applied for silencing RANK signaling using thermosensitive chitosan hydrogel as siRNA reservoir and vector.

**Results:**

The thermosensitive chitosan hydrogel was formed from solution (PH = 7.2, at 4°C) at 37°C within 8 minutes. The degradation rates of hydrogel were ~50% and 5% (W remaining/W beginning) in the presence and absence of lysozyme, respectively, over a period of 20 days. The concurrent cumulative *in vitro* release of Cy3-labeled siRNA from the hydrogel was 50% and 17% over 14 days, with or without lysozyme digestion, respectively. High cell viability (>88%) was maintained for cells treated with hydrogel loaded with RANK specific siRNA and RANK knockdown was prolonged for up to 9 days when cells were incubated with siRNA/hydrogel complex. *In vivo* release of siRNA was investigated in a subcutaneous delivery setup in mice. The fluorescent signal from siRNA within hydrogel was remained for up to 14 days compared to less than one day for siRNA alone.

**Conclusions:**

Chitosan hydrogel can potentially serve as a suitable reservoir and vector for local sustained delivery of siRNA in potential therapy.

## Background

Most of periodontal diseases are highly destructive and characterized by loss of the periodontal ligament and alveolar bone, eventually causing tooth loss. It is possible to minimalize the disease progression to some extent via routine dental hygiene improvements but no traditional therapy can efficiently recover the reattachment of periodontal ligament tissues and induce the bone regeneration.

The alveolar bone damage is usually caused by abnormal activity of osteoclast. The receptor activator of NF-κB (RANK) and its ligand RANKL are key molecules for differentiation and activation of osteoclasts [1]. When RANKL binds to RANK, it triggers downstream signaling that results in transformation of precursor cells to mature osteoclast. It would therefore be desirable to inhibit the pathway of osteoclast maturation via targeting RANK for prevention of alveolar bone loss.

RNA-interference (RNAi) is a post-transcriptional gene silencing process that is mediated either by synthetic small interfering RNAs (siRNAs) or endogenous microRNAs. RNAi has been established as an efficient tool for investigation of gene function due to its high specificity of gene silencing including genes associated with many diseases [2]. Therapeutic RNAi applications have been well developed over the recent years and significant progress have been made in therapies for cancer [3], neurodegenerative diseases [4], infectious [5] and inflammatory lesions [6-8]. However, the main concern for siRNA therapeutics is the development of a safe delivery system for the siRNA that can protect siRNA from degradation and deliver it into specific cells without side effects. Among non-viral delivery systems, traditional lipid-based transfection system has been developed for decades and widely applied due to its well-controlled pharmacokinetic behavior and high transfection efficiency; however, the drawbacks are cytotoxicity and limited durability, which limit the application *in vivo *[9]. Chitosan is a natural cationic polymer that can bind negative charged siRNAs via an electrostatic interaction to form complexes spontaneously in slightly acidic aqueous milieu. It has been comprehensively investigated for drug, plasmid [10] and siRNA delivery [11,12], and considered a suitable transfection vehicle due to its low toxicity, low immunogenicity, low cost, and good biocompatibility [13]. We have also successfully applied chitosan/siRNA derived therapies for arthritis and radiation-induced fibrosis in animal disease models [7,8]. Hydrogel containing siRNA has also been demonstrated effective in cancer therapy [14] and chitosan-based hydrogel has been used as a reservoir for drug loading to enable sustained release of variously therapeutic agents at controlled positions [15,16]. In particular, a chitosan-glycerol phosphate thermosensitive hydrogel, with improved biocompatibility [17,18] and rapid solution-gel state shifting [19], has been employed to deliver ellagic acid [20], collagen composite [21] or ferulic acid [22] for the treatment of various diseases including cancer [23]. In our study the chitosan hydrogel is loaded with RANK siRNA with aim to develop potential RNAi- based therapeutic strategy for periodontitis.

## Results

### siRNA Validation

Three siRNAs, designed to target mouse RANK, were transfected in murine macrophage cell line, RAW264.7. Two of these siRNAs showed a strong knockdown of RANK mRNA expression (Additional file 1: Figure S1). siRNA2 was selected for further experiments and referred as siR-RANK.

### Hydrogel mediated prolonged gene silencing

**Figure 1:**
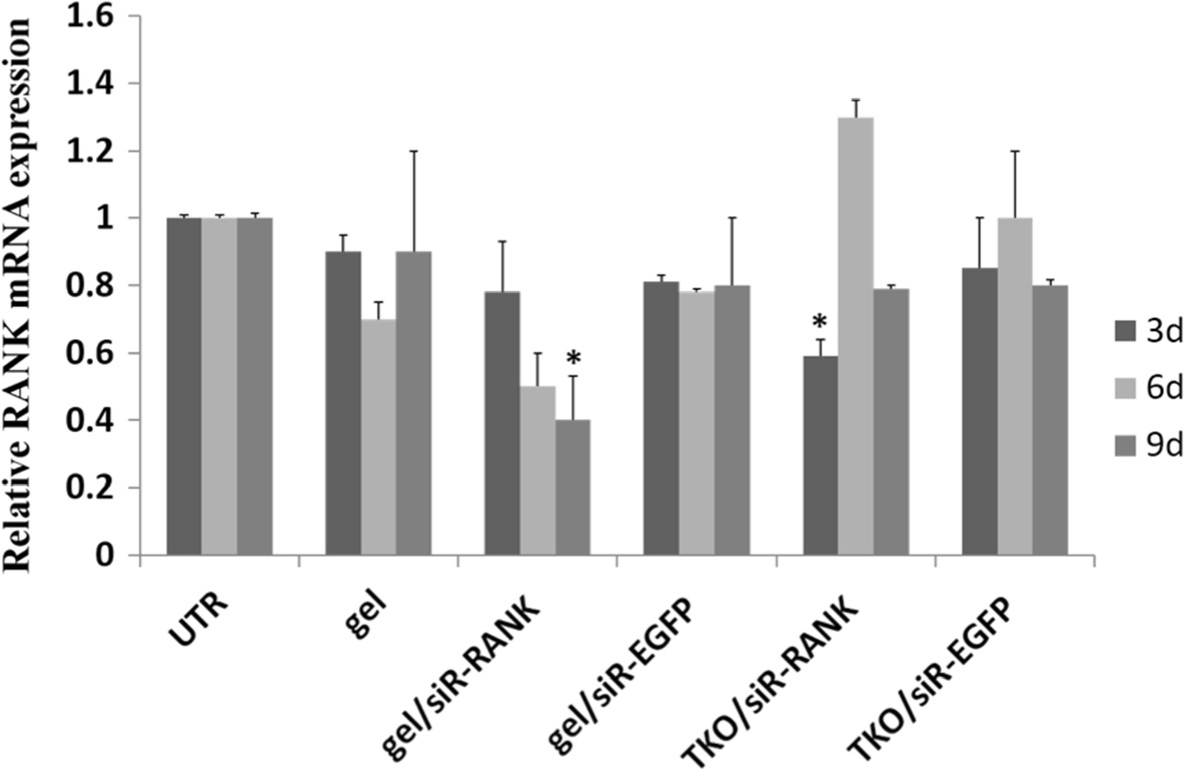
**Prolonged gene silencing by siRNA/hydrogel complex.** Cells were treated by hydrogel loaded with siR-RANK for 3, 6, and 9 days. Cells transfected via TransIT TKO/siR-RANK was included as positive control, and other controls included untransfected cells (UTR), empty hydrogel (Gel), or hydrogel formulated with siR-EGFP and TKO/siR-EGFP. The knockdown efficiency was evaluated by Real Time RT-PCR. RANK mRNA expression was normalized with β-actin and the relative RANK mRNA expression was calculated with UTR set to 1 and the data was presented as mean ± SD (n = 3). *p < 0.05 compared to untransfected cells.

The bioactivity of hydrogel loaded with siR-RANK was investigated in RAW264.7 cells. The efficiency of RANK gene silencing was evaluated at day 3, 6 and 9 post-transfection. Transfection with siRNA formulated using the commercial reagent TransIT-TKO was included as positive control for transfection efficiency. Compared to the >70% knockdown effect 48 h post transfection by TKO (Additional file 1: Figure S1), the RANK mRNA expression was partly recovered after 72 h (3 days) (Figure 1) (>40%), and completely returned to untreated level 6 and 9 days post transfection (Figure 1). In contrast, cells treated with hydrogel loaded with siR-RANK demonstrated a significant prolonged RANK mRNA knockdown effect gradually increasing from 30%, 50% to 60% at 3 d, 6 d and 9 d post transfection, respectively (Figure 1), with statistical significance of p < 0.05 (evaluated by one-way ANOVA).

### Cell viability assay

**Figure 2:**
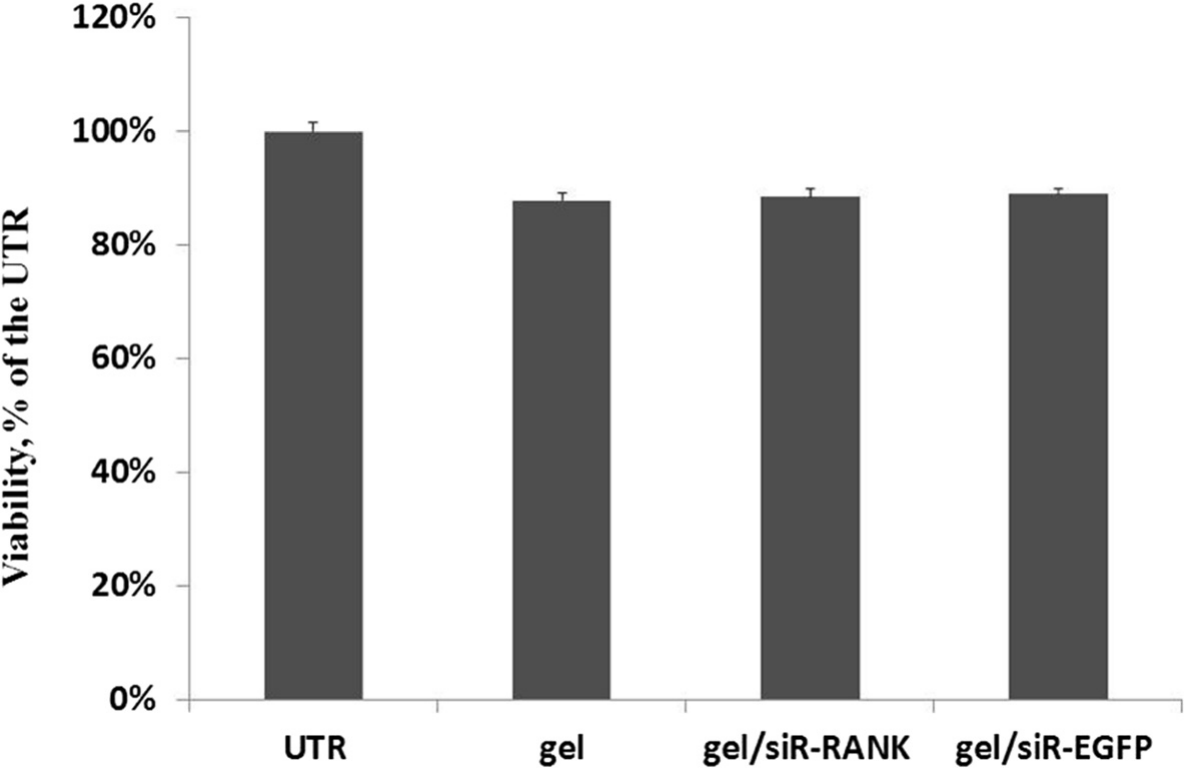
**Viability for cells incubated with chitosan hydrogel alone or coupled with siRNA.** Cell viability was assessed by MTT assay at 72 h post-transfection. The relative absorbance (at 570 nm) was measured and normalized to the level of non-treated control. Data was presented as mean ± SD (n = 3).

To evaluate the cytotoxicity of the hydrogel alone or hydrogel/siRNA complex, MTT assay was performed for cell treated with hydrogel and chitosan/siRNA hydrogel for 3 days (Figure 2). Compared to the non-treated control, viability of cells incubated with chitosan hydrogel alone or formulated with siRNAs was ~90%, indicating good biocompatibility and relatively low cytotoxicity of the hydrogel with and without siRNA.

### siRNA release test *in vitro*

**Figure 3:**
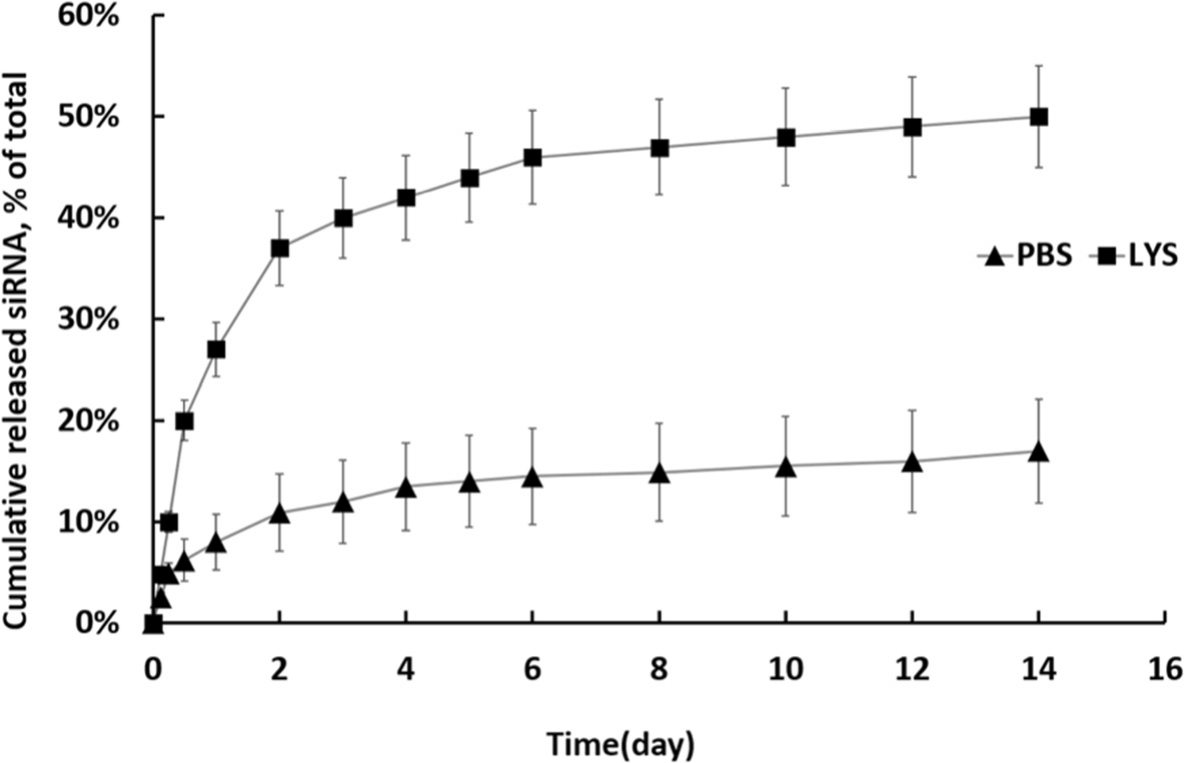
**Cumulative release profile of siRNA from chitosan hydrogel/siRNA.** The formulated hydrogel was degraded either in PBS alone (marked as PBS) or PBS contained lysozyme (marked as LYS). Fluorescence intensity was measured for samples collected from various time points and background level from blank well was subtracted. The accumulative release profiles were calculated based on concentrations obtained. Data was presented as mean ± SD (n = 3).

In order to determine the release profile of siRNA from chitosan hydrogel, Cy3-labeled siRNA was formulated with the hydrogel and subsequently incubated at 37°C in PBS. As a reference, a fluorescence intensity-concentration standard curve was determined with multiple dilution of Cy3-labeled siRNA in PBS and PBS alone. Fluorescence intensity was measured for samples collected from various time points and background signal from PBS alone was subtracted. The accumulative release profiles were calculated based on the concentrations obtained (Figure 3). A biphasic release profile was observed: A low initial burst for the first 2–3 days followed by a sustained slow release with a total of 17% released siRNA at day 14. The siRNA was released much faster when the hydrogel was treated with lysozyme, accumulating to a total of 50% release at day 14 (Figure 3).

To investigate whether siRNA is released as free siRNA or siRNA complexed with chitosan, a filter assay was performed. As expected, free siRNA, with a molecular weight of 13.3 kD, passes nearly completely through a membrane with MWCO of 100 kDa (Additional file 2: Figure S2). In contrast, less than 10% siRNA was recovered in the filter assay when formulated with chitosan indicating that the siRNA remains bound positively charged chitosan after release from the hydrogel.

### Hydrogel degradation *in vitro*

**Figure 4:**
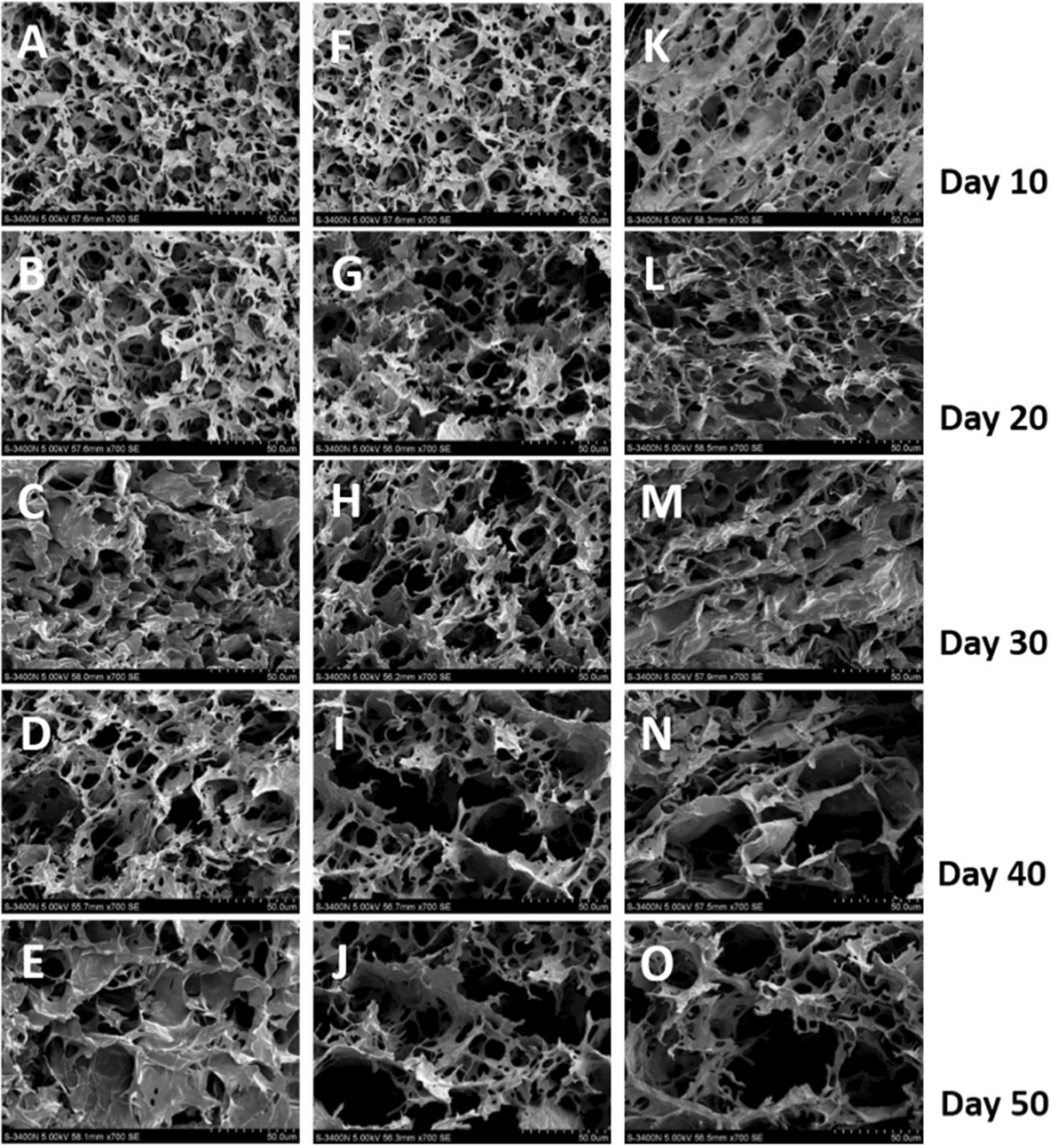
**FE-SEM images of hydrogel.** Hydrogel incubated in the absence **(A-E)** or presence of, 0.1 g/L and 0.5 g/L lysozyme (**F-J** and **K-O**, respectively; scale bar = 50 μm).

**Figure 5:**
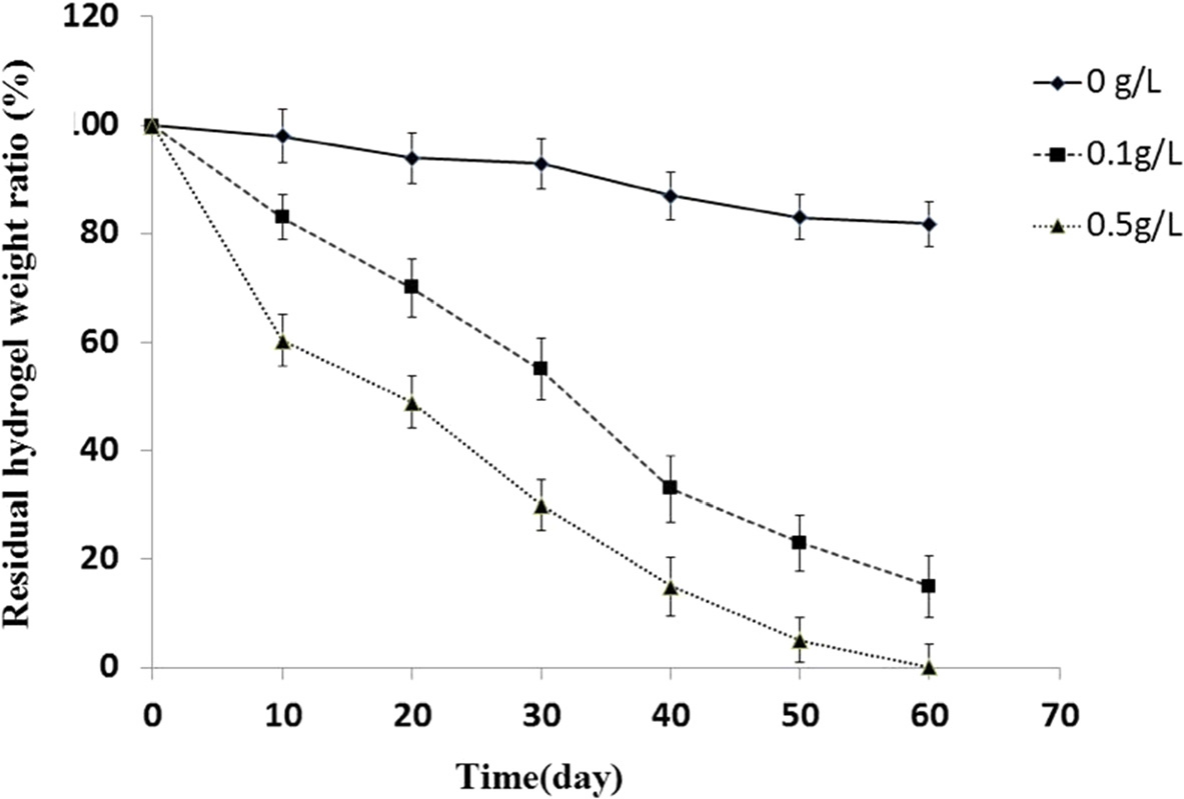
**Mass loss of hydrogel treated by lysozyme.** Hydrogel was incubated in the absence or presence of lysozyme at various concentrations (0.1 g/L and 0.5 g/L), and the residual weight of dried hydrogel was measured at different intervals. Data were presented as mean ± SD (n = 3).

To understand the degradation behavior of the hydrogel material, FE-SEM imaging was used to visualize the gel structure at different time points after gel-formation. Surface images showed that the non-treated hydrogel formed a complicated sponge-like structure with abundant cross-linking and the average diameter of pores of approximately 50 m (Figure 4A-E). The lysozyme treatment changed the apparent structure dramatically (Figures 4F-J and 5K-O), especially in the presence of 0.5g/L of lysozyme at day 10. Here the frame structure was loosened during the incubation and the cross-linking was destroyed, resulting in enlarged cavities (Figure 4K vs. 4A and F). No significant difference was observed between 0.1g/L and 0.5 g/L after extended incubation (>20 days) (Figure 4G-J and 5I-O). To quantify the hydrogel degradation, the residual chitosan hydrogel was weighed at different time points. The lysozyme clearly enhanced the degradation rate and the mass loss showed a positive correlation with the concentration of lysozyme (Figure 5). It is reasonable to speculate that the initial burst of chitosan/siRNA hydrogel in PBS may derive from surface associated siRNA by simple diffusion, while the prolonged release might involve interiorly located siRNA depending on the hydrogel degradation for release.

### siRNA release test *in vivo*

**Figure 6:**
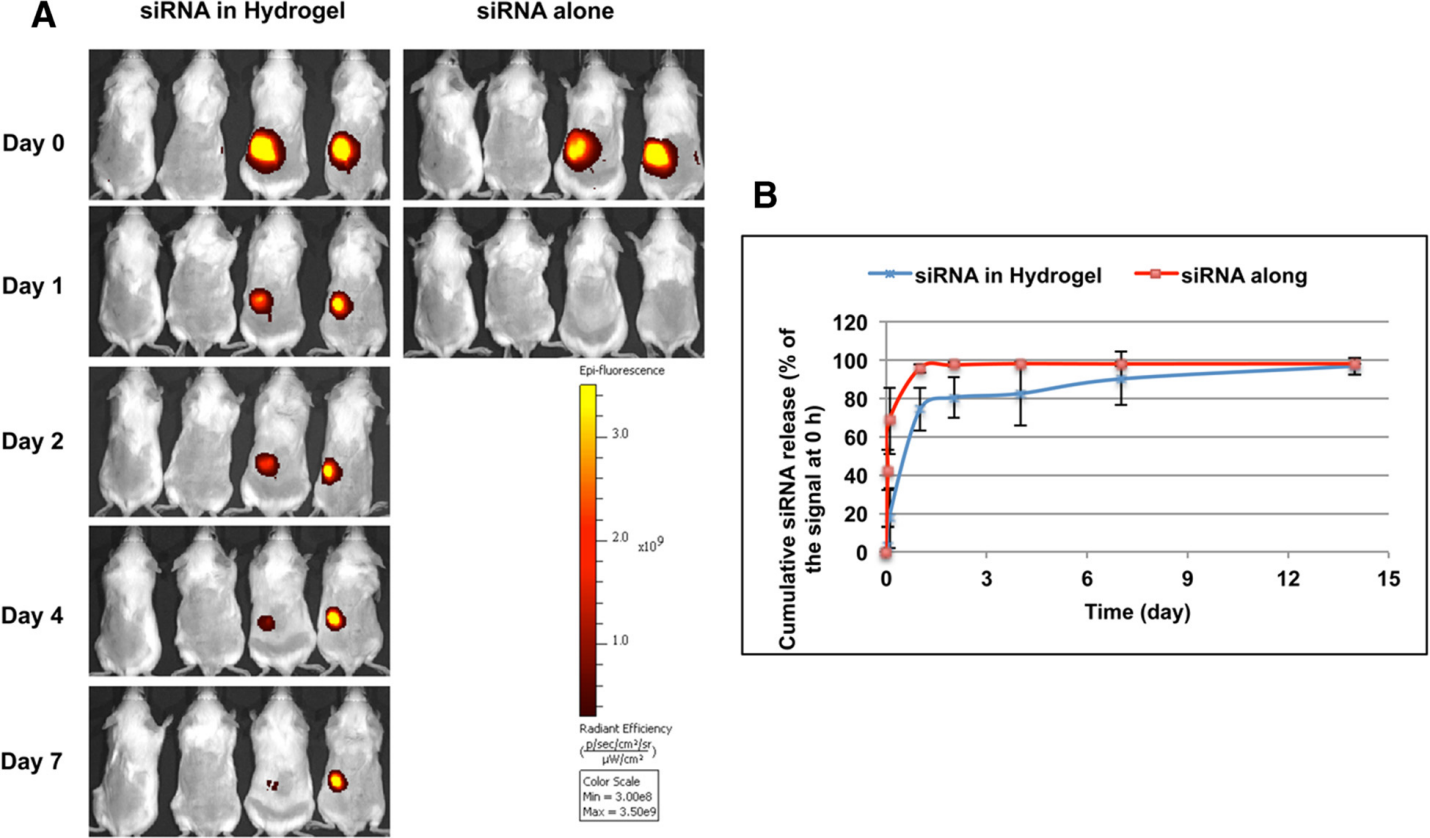
**Release profile of Cy5 labeled siRNA complexed within hydrogel in mice.** Mice were injected s.c. with chitosan hydrogel/Cy5-siRNA, Cy5-siRNA alone and PBS buffer (n = 4). Fluorescent optical imaging was performed at the indicated time points after injection. **(A)**, Images from 2 mice 0, 1, 2, 4 and 7 days after injection with chitosan hydrogen/Cy5-siRNA and images from 2 mice 0 and 1 day after injection with Cy5-siRNA alone. Two mice injected with buffer (Two mice on the left in each panel) were included as control for both scanning. **(B)**, After quantifying the signal intensity of images, siRNA release was calculated as reduction of fluorescent signal: (RE (0 h) – RE (Designed Time Point)) /RE (0 h) * 100%. Average value from each time point (0, 1, 2 h, and 1, 2, 4, 7, 14 d) was presented (mean ± SD, n = 4).

*In vivo* release of siRNA was investigated by optical *in vivo* imaging after subcutaneous injection of Cy5-siRNA loaded hydrogel in mice. Injection of siRNA alone in PBS was included as control. The fluorescent signal from siRNA within hydrogel remained for up to 7 days compared to less than one day for siRNA alone (Figure 6A). A quantitative release profile was provided by measuring the fluorescent signal using Living Image 4.2 Software (Figure 6B). A more sustained siRNA signal was demonstrated for mice injected with siRNA loaded in hydrogel than from mice injected with siRNA alone (~70% *vs* >95% decrease in signal intensity 24 h post-injection, respectively; Figure 6B).

## Discussion

We have demonstrated that siRNA, loaded into chitosan hydrogel, is released in a sustained fashion both *in vitro* and *in vivo*. Using this system, RANK specific siRNA exhibited a prolonged specific silencing effect for up to 9 days *in vitro* without apparent cytotoxicity.

RNAi has recently proven a promising approach for the development of new treatment protocols for human diseases. However, the lack of suitably non-viral systemic delivery vehicles has limited its application in clinic where effective and prolonged silencing effects are needed. Compared to systemic delivery, local delivery systems can provide a relatively high drug concentration in the lesion areas and reduce side effects. For instance, local delivery of siRNA using biodegradable polymer enabled a scaffold to support and repair bone defects [24]. However, the predetermined shape restricts the application for many kinds of lesions encountered in clinic. To address this, injectable hydrogel is widely applied for tissue engineering, also for drug delivery including siRNA delivery [25-27], due to its ability to undergo sol–gel phase transition.

We have successfully applied chitosan/siRNA therapies in several animal disease models with arthritis and radiation-induced fibrosis [7,8]. Among chitosan-based hydrogels, the one cross-linked with β-glycerophosphate has been most extensively employed and already proven effective in several kinds of drug delivery [20-22,25]. In addition, the chitosan-based gels have been used for tissue regeneration in periodontal disease [28]. We have developed an injectable thermosensitive chitosan hydrogel system for siRNA delivery and observed prolonged knockdown efficacy for up to 9 days. The effect is more pronounced than in a previous collagen gel study where substantial knockdown of GFP was observed for only up to 6 days in GFP expressing HEK293 cells [26].

The *in vitro* siRNA release profile suggests that electrostatic interactions between the nucleotides and positively charged polymer slow down the release. In addition, diffusion through the biopolymer pores facilitated by biopolymer degradation may also control the release. This resembles the observation in a previous report for siRNA release from calcium cross-linked alginate with chitosan [26]. In that study siRNA could only be released from the hydrogel when gradually degraded by an enzyme [26]. In another study, gelatin hydrogel, incorporated with cationized gelatin (CG)-siRNA complexes, was enzymatically degraded by the treatment with collagenase to enable the release of siRNA [25]. Similarly, we observed clear degradation in the presence of lysozyme, indicating that this enzyme is able to degrade the chitosan gel *in vitro *[29-31].

Compared to *in vitro*, siRNA was released more quickly *in vivo*, implying the existence of lysozyme or other degradation pathways *in vivo *[32]. The chitosan hydrogel/siRNA system has previously been applied in tumor therapy in mice [27]. They observed labelled siRNA uptake in tumour cells up to 270 μm away from the hydrogel edge 24 h post intra-tumoral injection, however, the half-life of siRNA *in vivo* was not addressed. The hydrogel was administrated twice weekly at a dose of 150 μg/kg body weight and resulted in significant antitumor effect [27]. Similarly, the biodegradable hydrogel, poly-D, L-lactic acid-p-dioxanonepolyethylene glycol block co-polymer (PLA-DX-PEG) has been used as a carrier for Noggin specific siRNA *in vivo*, resulting in efficient silencing of the target mRNA expression and successfully induces the ectopic bone formation [24].

The RANK-RANKL system has been proven to play a vital role in the differentiation and activation of osteoclasts in periodontitis and silencing the RANK expression in osteoclast precursors may provide a new approach to prevent and convert the alveolar absorption in periodontitis. In this study, we demonstrate chitosan hydrogel could prolong gene silencing effect of siRNA up to 9 days *in vitro*. Theoretically, this extended release profile may be provide sufficient siRNA to prevent activation of osteoclasts and maintain the therapeutic effects on periodontitis for an extended period of time. In combination with the excellent biocompatibility we observed, our system may potentially serve as a suitable vector for continuous local delivery of RANK siRNA in periodontitis therapy.

## Conclusions

In this study we have developed a chitosan-glycerol phosphate thermosensitive hydrogel enabling to functionally deliver RANK siRNA into cells. The hydrogel exhibited prolonged siRNA release profiles both *in vitro* and *in vivo*, accompanied with sustained gene silencing effect in cell. The next step will be to investigate its efficacy in animal periodontitis model and explore its potential to regenerate periodontal tissues.

## Methods

### siRNA target RANK screening

Three siRNA duplexes targeting RANK (siRNA1, siRNA2, and siRNA3) were ordered from GenePharma (Shanghai, China). The negative control siRNA (siNC) was provided by Genepharm, and siRNA-EGFP duplex was purchased from Ribotask (Odense, Denmark). All the sequences were listed in Table 1.

**Table 1:** Sequence of siRNAs

**Target**	**Sense sequence (5′-3′)**	**Antisense sequence (5′-3′)**
RANK1	GAGCAGAACUGACUCUAUGUU	CAUAGAGUCAGUUCUGCUCUU
RANK2	GCGCAGACUUCACUCCAUAUU	UAUGGAGUGAAGUCUGCGCUU
RANK3	GCGCUGACAGCUAAUUUGUTT	ACAAAUUAGCUGUCAGCGCTT
EGFP	GACGUAAACGGCCACAAGUTC	ACUUGUGGCCGUUUACGUCGC
Negative control	UUCUCCGAACGUGUCACGUTT	ACGUGACACGUUCGGAGAATT

Mouse macrophage cell line, RAW264.7 (ATCC® TIB-71™), used as osteoclast precursor, was maintained in RPMI media (Gibco®) supplemented with 10% fetal bovine serum (Sigma), 1% penicillin-streptomycin (Gibco®) at 37°C in 5% CO2 and 100% humidity. Cells were seeded onto 24-well plate twenty-four hours before transfection. siRNA was transfected using the commercial reagent TransIT-TKO (Mirus Bio Corporation) at final siRNA concentration 50 nM. Cells were harvested at 48 hr post-transfection.

mRNA expression level was assessed by Quantitative Real-Time RT-PCR (qRT-PCR) following routine procedures of total RNA purification using TRIzol reagent (Invitrogen, Copenhagen) and reverse transcription using SuperScript® *II Reverse Transcriptase* (RT) kit (Invitrogen, Copenhagen). The comparative CT (threshold cycle) method described in the manufacturer’s protocol (Stratagene, Copenhagen) was used to quantitate the relative RANK mRNA expression level. The β-actin mRNA was amplified as an internal control to normalise the expression level. The primer sequences for RANK gene are, forward: 5’-TAGGACGTCAGGCCAAAGGACAAA -3’, reverse: 5’-AGGGCCTACTGCCTAAGTGTGTTT -3’, with a product size of 132 bp. Primer sequences for β-actin gene are, forward: 5’-ACACAGTGCTGTCTGGTGGT-3’, Reverse: 5’-CTGGAAGGTGGACAGTGAGG-3’ with a product size of 172 bp.

### Formulation of chitosan hydrogel /siRNA complex

Chitosan (150 kDa, 95% deacetylation) was provided by HEPPE MEDICAL (Germany). Indicated chitosan was dissolved in 1% acetic acid (Sigma) at the concentration of 2% w/v. Sodium glycerophosphate (Sigma) were used as crosslink reagent and added dropwise into chitosan solution while stirring (pH 7.2, 4°C). Pre-calculated siRNA (5 μg) were added into the clear and homogeneous liquid solution (200 μl). The hydrogel spontaneously became solid during the incubation at 37°C for 8 minutes, which was ready for further experiments.

### siRNA transfection mediated by hydrogel

**Figure 7:**
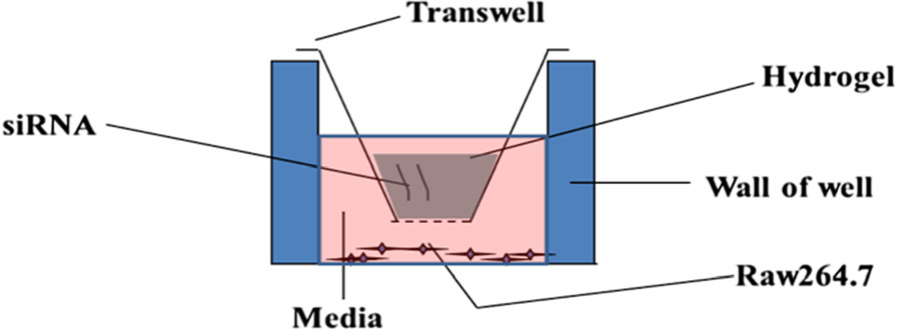
Relative position of chitosan/siRNA hydrogel in transwell during cell culture.

The 24-well transwell ( Thincert, In Vitro As, Fredensborg, Denmark) loaded with hydrogel was inserted over the cell culture plates where containing pre-seeded cells (RAW264.7, 2 × 10^4^/well), as shown in Figure 7. The transfection was terminated and the RANK gene expression was measured by Quantitative Real Time RT-PCR after transfected for 3, 6, 9 days.

### Cytotoxicity of hydrogel/siRNA complex

The cell viability was determined by MTT assay at day 3 of hydrogel mediated transfection as described above (n = 3). MTT reagent (5 mg/ml, Sigma, Copenhagen) was then added to each well at 1:3 dilution, after removed transwells, rinsed with PBS and exchanged the medium. After incubation of 2 ~ 4 h at 37°C, the reaction was stopped by replacing of DMSO (Sigma, Copenhagen) while the color changes became visible. The relative absorbance (at 570 nm) was measured using a Victor X5 Multilabel Plate reader (PerkinElmer).

### siRNA release test in vitro

To assess siRNA release profile, the Cy3-labled siRNA (5 μg) was formulated with chitosan hydrogel as described above. The hydrogel was immersed in 500 μl phosphate buffer (PBS, Gibco®) solution (pH 7.4) with or without lysozyme (0.5 mg/ml, Sigma). At pre-determined intervals, all of the supernatant was drawn and replaced by the fresh buffer accordingly. The fluorescence intensity from the supernatant was measured by fluorescence plate reader (FLUOstar OPTIMA) and PBS solution was used as blank control.

To investigate whether siRNA in release solution as free or chitosan complexed siRNA, Cy3-labled siRNA was encapsulated in chitosan hydrogel (n = 3). After gelation, 500 μl of PBS was added and incubated 2 h at 37°C. The PBS solution was collected and 250 μl the solution was loaded on to a centrifugal device with molecular weight cut off (MWCO) of 100 kDa (Pall Corporation) and centrifuged at 3000 g for 5 min. The Cy3 fluorescent intensity of filtered solution was measured by FLUOstar OPTIMA (BMG Labtechnologies) and normalized to input solution. As a non-particulate control, free Cy3-siRNA was also loaded on a centrifuge device treated similarly.

### Hydrogel degradation *in vitro*

To further understand the mechanism of siRNA release from the hydrogel, the hydrogel degradation was assessed. Briefly, 1ml chitosan hydrogel solution was placed into 37°C incubator to form the hydrogel. The hydrogel, treated using three concentration of lysozyme (0, 0.1, 0.5 g/L), was quickly frozen with liquid nitrogen for 5min and totally freeze-dried before precisely weighed by electronic balance. The percentage of mass loss was determined by measuring hydrogel weight at initial time point (Wi) and weight at different time points pre-designed (Wd), and calculated by ((Wi-Wd)/Wi). After gold coating, the freeze-dried sample was also observed with Field Emission Scanning Electron Microscope (FE-SEM).

### siRNA release test *in vivo*

To evaluate siRNA release within hydrogel *in vivo*, BALB/c female mice (Taconic Europe, Ll. Skensved, Denmark) were chosen. All procedures of animal work were approved by “The Experimental Animal Inspectorate in Denmark” under The Danish Veterinary and Food Administration, Ministry of Food, Agriculture and Fisheries.

Hair from the back and the abdomen was shaved to avoid autofluorescence during scanning. Cy5-labelled siRNA/chitosan hydrogel formulation in 50 microliter sodium acetate buffer (containing 5 μg siRNA duplex) was subcutaneously injected into the left flank region of mice. The unformulated Cy5-labelled siRNA in PBS with the same volume was administrated as control. The mice were scanned using an IVIS® 200 imaging system (Xenogen, Caliper Life Sciences) before and right after injection followed by 1 hr, 2 hrs, 1 day, 2, 3, 7 and 14 days post injection. The scanning was performed under anesthesia with 2.5% isoflurane. Cy5 excitation (λex = 640 nm) and emission (λem = 700 nm) filters were used. Total emission from inflicted areas (Region of Interest, ROI) of each mouse was quantified using Living Image 4.0 software package. According to the user instruction, the radiant efficiency (RE) of injection sites was measured (photons/sec/cm2/sr)/ (μW/cm2), which is presented radiance/illumination power density [33,34].

## Competing interests

The authors declare that they have no competing interests.

## Authors’ contributions

ZM designed and carried out all the experiments. CY carried out the hydrogel preparation and joined most of experiments. WS analyzed the data and drafted the manuscript. QW participated in the design of the study. JK analyzed the data and revised the manuscript. SG drafted and revised the manuscript. All authors read and approved the final manuscript.

## Supplementary Material

Additional file 1: Figure S1 RANK gene knockdown efficiency. Three siRNA against murine RANK (siRNA1, siRNA2 and siRNA3) were transfected in triplicate into RAW264.7 cells using TransIT-TKO reagent. Untreated cells (UTR) or cells transfected with siRNA against EGFP (siR-EGFP) and siR-NC were included as controls. Cells were harvested 48 hrs post transfection and RANK mRNA levels were evaluated by quantitative RT-PCR. Data were presented as mean ± SD (n = 3). * siRNA was selected for further experiment. Click here for file

Additional file 2: Figure S2 Analysis of siRNA released from chitosan hydrogel. The Cy3-labled siRNA was encapsulated in chitosan hydrogel (n = 3) and incubated in PBS for 2h at 37°C, the PBS solution was collected and half of the solution (release solution) was centrifuged through a filter device. The Cy3 fluorescent intensity of filtered solution was measured and normalized to input solution. Unformulated free Cy3-siRNA (free siRNA) was applied as control. Click here for file
