# Supplementary figures and images for: Chitosan Hydrogel as siRNA vector for prolonged gene silencing
Source: J Nanobiotechnology. 2014 Jun 19;12:23. doi: 10.1186/1477-3155-12-23 (PMC4104730; doi:10.1186/1477-3155-12-23)

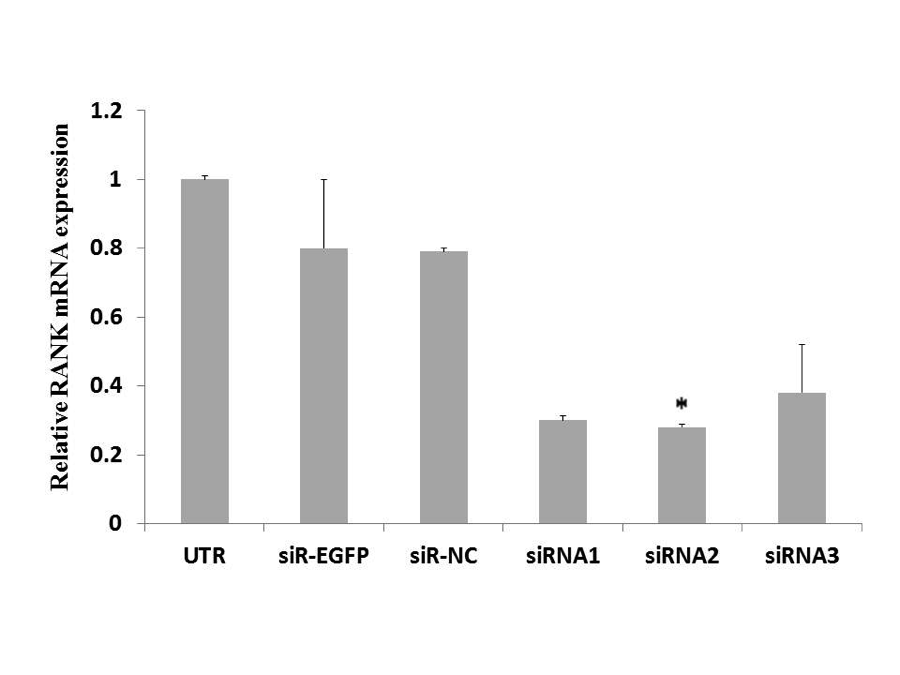

Supplement: Additional file 1: Figure S1 — RANK gene knockdown efficiency. Three siRNA against murine RANK (siRNA1, siRNA2 and siRNA3) were transfected in triplicate into RAW264.7 cells using TransIT-TKO reagent. Untreated cells (UTR) or cells transfected with siRNA against EGFP (siR-EGFP) and siR-NC were included as controls. Cells were harvested 48 hrs post transfection and RANK mRNA levels were evaluated by quantitative RT-PCR. Data were presented as mean ± SD (n = 3). * siRNA was selected for further experiment. [file 1477-3155-12-23-S1.png]

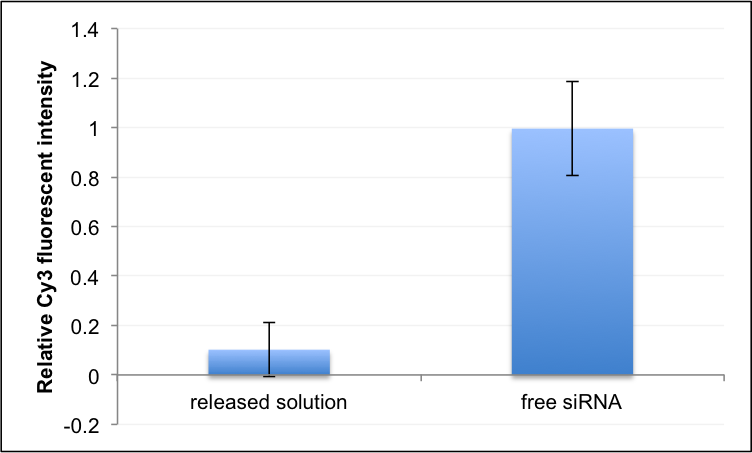

Supplement: Additional file 2: Figure S2 — Analysis of siRNA released from chitosan hydrogel. The Cy3-labled siRNA was encapsulated in chitosan hydrogel (n = 3) and incubated in PBS for 2h at 37°C, the PBS solution was collected and half of the solution (release solution) was centrifuged through a filter device. The Cy3 fluorescent intensity of filtered solution was measured and normalized to input solution. Unformulated free Cy3-siRNA (free siRNA) was applied as control. [file 1477-3155-12-23-S2.png]
